# Outcomes of Primary Flexor Tendon Repairs in Zones 2 and 3: A Retrospective Cohort Study

**DOI:** 10.1016/j.jhsg.2023.03.016

**Published:** 2023-05-18

**Authors:** Bernadette Christina Tobler-Ammann, Vera Beckmann-Fries, Maurizio Calcagni, Alexandre Kämpfen, Lorena Schrepfer, Esther Vögelin

**Affiliations:** ∗ Department of Plastic and Hand Surgery, Inselspital Bern, University of Bern, Bern, Switzerland; † Department of Physiotherapy and Occupational Therapy, University Hospital Zurich, Zurich, Switzerland; ‡ Department of Plastic Surgery and Hand Surgery, University Hospital Zurich, Zurich, Switzerland; § Department of Plastic, Reconstructive, Aesthetic and Hand Surgery, University Hospital Basel, Basel, Switzerland

**Keywords:** Clinical outcomes, Cohort, Flexor tendon injury, Zone 2, Zone 3

## Abstract

**Purpose:**

The aims of this retrospective cohort study were to present outcomes of zone 2 and 3 primary flexor tendon repairs and to evaluate how clinical outcomes change over time within and between zones of injury at weeks 6, 13, and 26.

**Methods:**

Data were retrieved from a multicenter flexor tendon cohort registry from 2014 to 2021. The inclusion criteria were: (1) adult patients after primary flexor tendon surgery in zone 2 or 3, (2) flexor digitorum profundus laceration of >50%, (3) 4–6 multistrand flexor digitorum profundus core suture, and (4) early active motion protocol. The primary outcome was the range of motion. Secondary outcomes were strength, patient satisfaction on an 11-point Likert scale, and self-reported physical function measured with the Disability of the Arm, Shoulder, and Hand questionnaire 6, 13, and 26 weeks after surgery.

**Results:**

We evaluated 33 patients after 39 tendon repairs in zone 3 and 174 repairs in zone 2 of 163 patients. Range of motion significantly improved over time in both zones (*P* < .001 to .01). Between-group range of motion differences were nonsignificant except for week 26 (*P* < .001) for the zone 3 group. Hand strength significantly improved in both zones over time (*P* < .001 to .01), while between-zone strength differences were statistically nonsignificant (*P* = .37 to .93). Patient satisfaction was generally good to high (mean 6.8 to 8.0 points) with significant within-group changes in both zones (*P* < .001). There were no relevant between-zone differences in Disability of the Arm, Shoulder, and Hand scores at any time point.

**Conclusions:**

Patients had significantly improved clinical outcomes in both zones. The zone of injury significantly affected the total active motion scores at the final assessment after 26 weeks for the zone 3 injuries. For the secondary outcomes hand strength, patient satisfaction, and Disability of the Arm, Shoulder, and Hand scores, we discovered no significant between-group differences.

**Type of study/level of evidence:**

Therapeutic IV.

With an incidence of 33.2 injuries per 100,000 person-years, tendon injuries in the hand and wrist are common among all emergency department visits.^1^ The relative distribution of flexor tendon injuries observed in zones 1, 2, 3, 4, and 5 are16%, 43%, 10%, 2%, and 29%, respectively.^1^, ^2^, ^3^ Although most literature is dedicated to the management of flexor tendon lacerations in zone 2,^4^, ^5^, ^6^, ^7^, ^8^, ^9^ reports on zone 3 outcomes are relatively scarce.^3^^,^^10^, ^11^, ^12^, ^13^

Zone 2 is located between the proximal border of the A1 pulley and the insertion of the flexor digitorum superficialis (FDS) tendon.^14^ Zone 3 lies between the distal border of the transverse carpal ligament and the proximal edge of the fibro-osseous sheath.^15^ Both zones include neurovascular structures and the flexor digitorum profundus (FDP) and FDS tendons.^16^ Tendons in zone 3 move in a more spacious environment without the confines of the fibro-osseous tunnel, allowing for surgery on both FDP and FDS tendons, even using a bulky repair.^10^^,^^14^^,^^17^ In contrast, even a slight swelling of the tendon(s) in this tunnel can block zone 2 repair(s) and eventually lead to adhesions or even rupture.^14^^,^^18^

Furthermore, there is limited published information relating to zone 3 rehabilitation. It was reported that early flexion contractures at the proximal and distal interphalangeal (PIP and DIP) joints after zone 3 injuries seem to respond better to hand therapy and splinting than those after zone 2 lesions.^10^ Potential reasons for this difference are the bigger distance between repair and these joints and less tight compartments in zone 3.^10^

In light of these anatomical and pathophysiological differences in the flexor tendons in zones 2 and 3, zone 3 injuries seem to be more “forgiving” and, therefore, should achieve better clinical results than zone 2 injuries.^10^ The primary purpose of this study was to present the outcomes of zones 2 and 3 primary flexor tendon repairs. The primary outcome was the range of motion (ROM). Secondary outcomes were hand strength, patient satisfaction, and patient-rated physical function. The secondary purpose was to evaluate how these therapy outcomes change over time within and between zones of injury.

## Materials and Methods

### Study design

Data for this retrospective cohort study were retrieved from a multicenter registry of flexor tendon repairs in zones 1–3 from 2014 to 2021. The departments of hand surgery and therapy of three independent centers reached a consensus on data management, time points, and choice of assessments to treat finger flexor tendon injuries prior to the start of data collection. Ethical approval was obtained from the local ethics committee (BASEC-Nr. 2017-02267). The Strengthening the Reporting of Observational Studies in Epidemiology statement was used to report this study.^19^

### Patients

**Figure 1:**
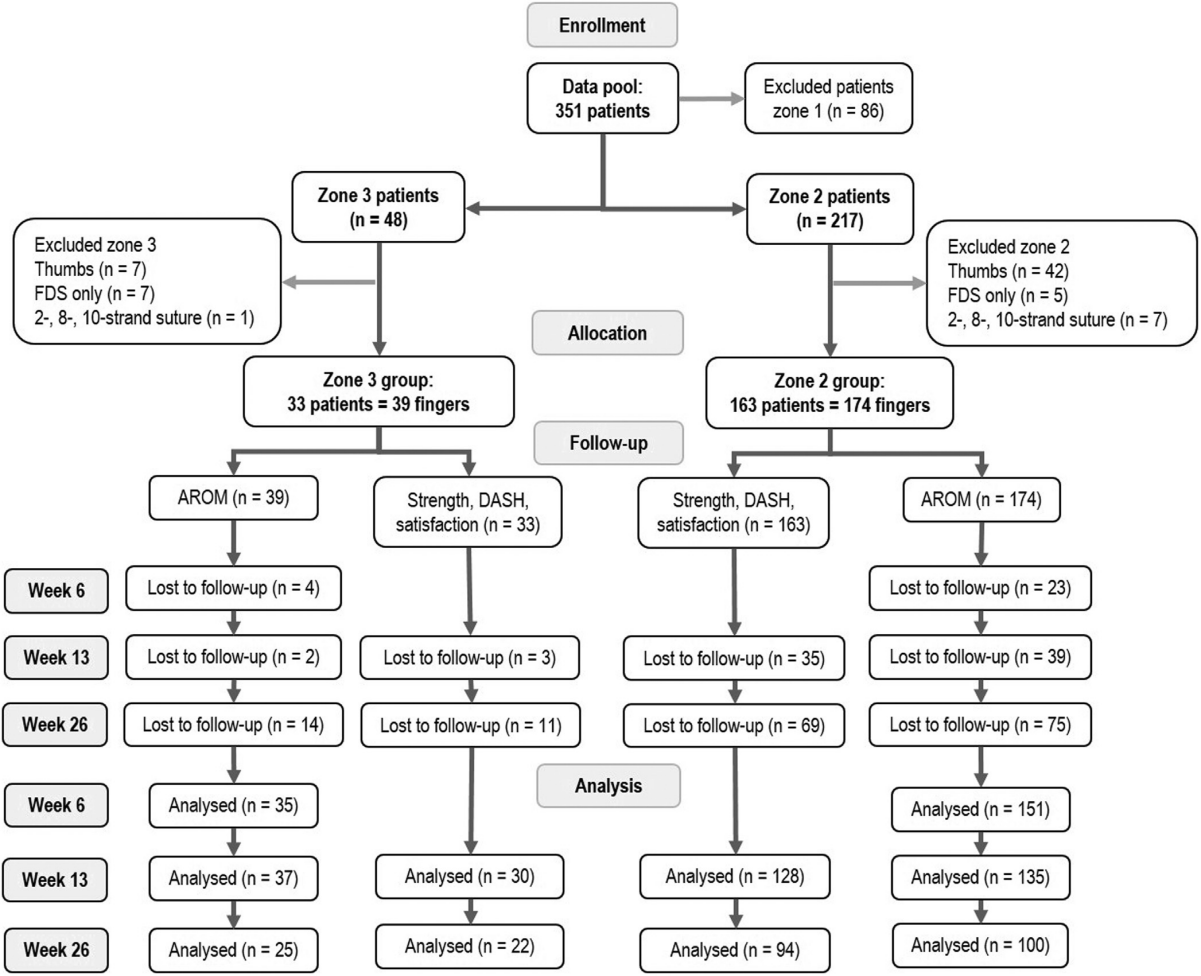
Study flow chart. AROM, active range of motion.

Patients in this registry were screened for the following inclusion criteria: (1) adult patients after primary flexor tendon surgery in zones 3 or 2, (2) an FDP laceration of ˃50% requiring surgery, (3) a 4–6 multistrand FDP core suture, and (4) an early active motion protocol. Exclusion criteria were: (1) flexor tendon injuries to the thumb, (2) zone 1 injuries, (3) FDP lesions ≤50% not requiring tendon surgery, (4) isolated FDS lacerations, and (5) complex concomitant injuries (fractures and amputations). The study flow chart (Fig. 1) shows the data collection process of the original data.

### Surgical technique

All patients underwent primary surgery in one of the three participating centers. For the repair of the FDP tendon, a 4 or 6-strand core suture, according to the Lim-Tsai, M-Tang, or Tsuge technique, was used for most lacerations. Seventy-five percent of zone 3 and 80% of zone 2 tendons had an additional circumferential suture. Supramid-Loop 4-0 and Supramid 4-0 were the most frequently used core suture materials. The FDS was sutured in 85% of all zone 3 tendon lacerations, whereas 37% of both the radial and ulnar slips were sutured in zone 2. Plexus anesthesia was most frequently chosen for surgery (50%), followed by general (29%) and local (21%) anesthesia. Pulleys were vented in 46% of the injured fingers in zone 3 and 58% in zone 2. The time-to-surgery from injury was on average 1.3 (SD 1.7) days in zone 3 and 3.0 (SD 4.8) days in zone 2. Because all participating centers are teaching institutions, the level of experience of the surgeon in training varied greatly. A senior surgeon always supervised the surgery. Hand therapists were all experienced in treating flexor tendon injuries.

### After surgery rehabilitation protocol

All patients were treated with an early active motion protocol in one of the hand therapy units of the three participating centers. Ninety percent of patients were treated following the controlled active motion (CAM) protocol and 10% according to the Manchester Short Splint (MSS) regimen.^20^, ^21^, ^22^ Patients received a long or short thermoplastic dorsal blocking splint within 3–5 days after surgery. In the CAM protocol, the wrist was positioned at 20° to 30° of extension. In the MSS protocol, the wrist was allowed to move freely until a maximum of 45° of extension. For both splints, metacarpal (MCP) joints were in 30° to 40° of flexion, and the interphalangeal joints were in 0°. The patients were instructed to wear their splints full-time for a period of 6 weeks.

Exercise emphasis was on training of passive full flexion of the fingers and gradually increasing active flexion of the fingers until full flexion at the end of week 3 without provoking discomfort in the form of tension or pain. Although patients in the MSS protocol were allowed to actively perform digital flexion exercises with the wrist extended to 45° and active digital extension exercises with the wrist in maximal flexion from the first week onward, these wrist tenodesis exercises were allowed out of the splint from week 4 onward in the CAM protocol. Patients in the MSS splint were allowed to use the injured hand for safe and light functional daily activities, excluding only the injured digit rather than the whole hand,^22^ whereas those in the CAM splints were instructed that the injured hand could be mobilized but not used for daily activities.^23^

### Outcome measurements

The primary outcome, ROM, was measured with a hand-held goniometer 6, 13, and 26 weeks after surgery. The minimal important difference (MID) ranges between 18° and 24° for the MCP, 12° and 15° for the PIP joints, and 14° and 18° for the DIP joints.^24^

The secondary outcomes of hand strength, patient satisfaction, and patient-rated upper-extremity disability were measured 13 and 26 weeks after flexor tendon injury. For hand grip strength, a Jamar Hydraulic Hand Dynamometer was used.^25^ The mean of three repetitions per hand was noted. The MID for grip strength was 5.0 to 6.5 kg.^26^

Patient satisfaction was assessed by asking the patient, “How satisfied are you with your hand today?” and rated on an 11-point Likert scale (0–10).^27^ The higher the score, the better the satisfaction. The MID ranged from 1.2 to 1.8 points.^27^ Any reasons for dissatisfaction were noted. Patient-rated upper-extremity disability and symptoms were evaluated using the Disability of the Arm, Shoulder, and Hand (DASH) questionnaire.^28^ Scores ranged from 0 to 100, and higher scores indicated greater disability. The MID after hand surgery of the DASH questionnaire ranged between 10.8 and 18.8 points.^29^

### Statistical analysis

A total of 99.5% of demographic data were available for analysis. Divided by zone of injury, 89.7% of primary outcome (ROM) data were available at 6 weeks for zone 3 and 86.8% for zone 2. For week 13, 94.9% of data were available for zone 3 and 77.6% for zone 2 patients. For week 26, 64.1% of the primary outcome data available were accessible for zone 3 and 56.9% for zone 2 (Fig. 1). Little test for missing data for the demographics was nonsignificant (*P* = .640), as well as for ROM outcomes at week 6 (zone 3, *P* = .117; zone 2, *P* ˃ .99) and week 13 (zone 3, *P* = .388; zone 2, *P* > .99), confirming that the data were missing completely at random. For week 26, Little test was significant (zone 3, *P* = .042; zone 2, *P* = .018). A nonresponder analysis was performed to further evaluate missing data at week 26, showing no significant differences between responders and nonresponders for both zones of injury (Supplementary Table 1, available on the *Journal’s* website at www.jhsgo.org). Therefore, missing data were replaced by multiple imputations.^30^ Means of the original and pooled data were controlled for accuracy (Supplementary Table 2, available on the *Journal’s* website at www.jhsgo.org).

Categorical data were reported as frequency and percentage, and continuous data were checked for normality and reported as the mean and standard deviation. Range of motion was calculated as follows: (1) the mean degrees per joint, (2) the total active (TAM) and passive (TPM) motion scores (calculated by subtracting the total active or passive flexion of the MCP, PIP, and DIP joints from the total active or passive extension deficit of the same joints), and (3) the percentage of return of motion compared to the contralateral side. We used two classification systems for results assessment after flexor tendon repairs. First is the one described by Tang^9^ where the MCP joint is not included in the calculation to meet the needs for zone 2 repairs. Second the one recommended by the American Society for Surgery of the Hand to meet the requirements of zone 3 injuries.^31^ Where measurements of the contralateral hand were missing, 175° or 260° was inserted, being the sum of motion of the normal PIP + DIP joints and MCP + PIP + DIP joints, respectively.^9^

Differences between measurement time points per zone of injury and between zones 2 and 3 were evaluated by a paired *t* test and independent-sample *t* test, respectively. Effect sizes were classified according to Cohen’s classification as small (r = 0.10), medium (r = 0.30), and large (r = 0.50).^32^ Level of significance was set at *P* ≤ .05.

As our study is retrospective, we did not perform a post hoc power analysis because the random component in the study disappears once data are collected, making such power estimates meaningless, as they cannot inform power for detecting significant results.^33^

## Results

Patients and injury characteristics were generally well matched between the two groups with some exceptions (Table 1, Table 2). For example, there were no ruptures or local anesthesia in zone 3 compared with 18 ruptures and 25% of patients with local anesthesia in zone 2. Of those patients with a tendon rupture, only demographic data were analyzed, as all ruptures occurred before week 6 (Table 1). Zone 3 patients did not receive an MSS splint because this regimen is designed for zone 1 and 2 injuries.

**Table 2:** Injury and Surgery Characteristics at Finger-Level∗

Variables	Overall	Zone 3	Zone 2	*P* Value
	n = 213	n = 39	n = 174	*P* ≤ .05
Injured fingers				
Digit 2	67 (31.5)	9 (23.1)	58 (33.3)	.21
Digit 3	33 (15.5)	6 (15.4)	27 (15.5)	.82
Digit 4	40 (18.8)	13 (33.3)	27 (15.5)	<.001†
Digit 5	73 (34.3)	11 (28.2)	62 (35.6)	.61
FDP complete laceration				
Digit 2	57 (85.1)	7 (77.8)	50 (86.2)	.51
Digit 3	32 (97.0)	6 (100)	26 (96.3)	.64
Digit 4	32 (80.0)	10 (76.9)	22 (81.5)	.74
Digit 5	71 (97.3)	11 (100)	60 (96.8)	.55
FDS complete laceration				
Digit 2	35 (52.2)	7 (77.8)	28 (48.3)	.13
Digit 3	18 (54.5)	6 (100)	12 (44.4)	.02‡
Digit 4	7 (17.5)	0	7 (25.9)	<.001‡
Digit 5	35 (48.0)	10 (83.3)	25 (40.3)	.01‡
FDS intact				
Digit 2	14 (20.9)	1 (11.1)	13 (22.4)	0
Digit 3	12 (36.4)	0	12 (44.4)	0
Digit 4	11 (27.5)	1 (7.7)	10 (37.0)	0
Digit 5	28 (38.4)	1 (9.1)	27 (43.5)	0
Nerve involved				
Digit 2	40 (59.7)	5 (55.6)	35 (60.3)	.79
Digit 3	13 (39.4)	4 (66.7)	9 (33.3)	.14
Digit 4	26 (65.0)	8 (61.5)	18 (66.7)	.75
Digit 5	26 (35.6)	5 (45.5)	21 (33.9)	.46
Joint involved				
Digit 2	5 (7.5)	0	5 (8.6)	.36
Digit 3	4 (12.1)	1 (16.7)	3 (11.1)	.71
Digit 4	4 (10.0)	2 (15.4)	2 (7.4)	.44
Digit 5	5 (6.8)	2 (18.2)	3 (4.8)	.12
Muscle injury				
Digit 2	5 (50.0)	5 (50.0)	0	0
Digit 3	3 (42.9)	3 (42.9)	0	0
Digit 4	7 (53.8)	7 (53.8)	0	0
Digit 5	6 (50.0)	6 (50.0)	0	0
Pulley involved, n				
Digit 2 (A1, A2, A3, A4)	15	0 (A1)	2, 2, 7, 4	-.64
Digit 3 (A1, A2, A3, A4)	12	0 (A1)	3, 1, 2, 6	-.78
Digit 4 (A1, A2, A3, A4)	8	0 (A1)	0, 2, 3, 3	0
Digit 5 (A1, A2, A3, A4)	22	0 (A1)	3, 5, 6, 8	.66
Pulleys				
Repair	21 (10.0)	1 (2.7)	20 (11.5)	.10
Venting	118 (57.3)	17 (45.9)	101 (58.0)	.13
Tendon gliding test	119 (55.9)	20 (51.3)	99 (56.9)	.52
Number of FDP strands				.04‡
4-strand suture	35 (16.4)	2 (5.1)	33 (19.0)	
6-strand suture	178 (83.6)	37 (94.9)	141 (81.0)	
FDP suture technique				.78
Lim-Tsai	89 (42.0)	19 (48.7)	70 (40.2)	
M-Tang	73 (34.4)	9 (23.1)	64 (36.8)	
Tsuge	23 (10.8)	8 (20.5)	15 (8.6)	
Modified M-Tang	11 (5.2)	3 (7.7)	8 (4.6)	
Modified Kessler	9 (4.2)	0	9 (5.2)	
FDP core suture material				.43
Supramid-Loop 4-0	96 (46.4)	21 (58.3)	75 (43.1)	
Supramid 4-0	50 (24.2)	10 (27.8)	40 (23.0)	
Braun-Tendofil® 4-0	19 (9.2)	2 (5.1)	17 (9.8)	
Fiber-Wire-Loop 4-0	16 (7.7)	2 (5.1)	14 (8.0)	
Fiber-Wire 4-0	10 (4.8)	1 (2.8)	9 (5.2)	
FDP circumferential suture	168 (79.2)	29 (74.4)	139 (79.9)	.41
FDS handling radial†				
Suture	98 (46.0)	33 (84.6)	65 (37.4)	<.001‡
Resection	31 (14.6)	1 (2.6)	30 (17.2)	.02‡
Untreated	9 (4.2)	3 (7.7)	6 (3.4)	.24
FDS handling ulnar†				
Suture	97 (45.5)	33 (84.6)	64 (36.8)	<.001‡
Resection	26 (12.2)	1 (2.6)	25 (14.4)	.04‡
Untreated	14 (6.6)	3 (7.7)	11 (6.3)	.76
FDS suture material				.03‡
Supramid-Loop 4-0	37 (33.6)	17 (54.8)	20 (25.3)	
Supramid 4-0	16 (14.5)	8 (25.8)	8 (10.1)	
Prolene 4-0	21 (19.1)	4 (12.9)	17 (21.5)	
PDS	11 (10.0)	0	11 (13.9)	

∗ All values are n(%) unless specified otherwise.

† There was no distinction in radial and ulnar FDS handling of zone 3 injuries, only for zone 2 injuries. Therefore, the same FDS values are presented twice in the table.

‡ Statistically significant difference between zones (*P* ≤ .05). These variables were entered into the regression models.

**Table 1:** Demographic, Injury, and Therapy Characteristics at Patient-Level

Variables	Overall, n (%)∗	Zone 3, n (%)	Zone 2, n (%)	*P* Value
	n = 196	n = 33	n = 163	*P* ≤ .05
Mean age ± SD, (yrs)	36.2 ± 14.3	35.4 ± 12.6	36.4 ± 14.6	.95
Male	134 (68.4)	24 (72.7)	110 (67.5)	.56
Blue collar worker	116 (61.4)	18 (58.1)	98 (60.1)	.68
Return to work	147 (85.5)	28 (93.3)	119 (73.0)	.19
Employment				.10
(Self-)employed	157 (80.8)	29 (90.6)	128 (79.0)	
Retired/nonworking/student	37 (19.2)	3 (9.4)	34 (21.0)	
Injured hand				
Left	102 (52.0)	17 (51.5)	85 (52.1)	.70
Nondominant	99 (51.8)	16 (48.5)	83 (50.9)	.82
Injured single fingers				
Dig 2	63 (34.8)	8 (29.6)	55 (35.7)	.66
Dig 3	23 (12.7)	3 (11.1)	20 (13.0)	.26
Dig 4	29 (16.0)	8 (29.6)	21 (13.6)	.01†
Dig 5	66 (36.5)	8 (29.6)	58 (37.7)	.06
Single	181 (92.3)	27 (81.8)	154 (94.5)	.93
Multiple (2 fingers)	13 (86.7)	6 (18.2)	7 (4.3)	.01†
Multiple (3 fingers)	2 (13.3)	0	2 (1.2)	0
Mechanism of injury				.93
Clean cut	168 (85.7)	28 (84.8)	141 (86.5)	
Mild crush	18 (9.2)	4 (12.1)	15 (9.2)	
Moderate crush	8 (4.1)	1 (3.0)	7 (4.3)	
Concomitant injury of other fingers				
Tendon ≤50 %	7 (3.6)	2 (6.1)	5 (3.1)	.40
Nerve	10 (5.1)	4 (12.1)	6 (3.7)	.05†
Mean time from injury to surgery ± SD, (d)	2.7 ± 4.5	1.3 ± 1.7	3.0 ± 4.8	.02†
Complications				
Ruptures	18 (9.2)	0	18 (11)	.05†
Adhesions resulting in tenolysis	17 (8.7)	1 (2.9)	16 (9.8)	.21
Surgery after rupture	14	0	14 (77.8)	0
Reconstruction after rupture	6	0	6 (3.7)	0
Mean time to 2nd surgery ± SD, (d)	41.6 ± 27.8	0	41.6 ± 27.8	0
Mean time to tenolysis ± SD, (wks)	29.6 ± 14.3	39.1 ± 0.0	29.1 ± 14.6	0
Mean hand therapy ± SD				
Sessions until 13 weeks, (n)	16.0 ± 5.9	16.9 ± 5.8	15.8 ± 5.9	.27
Sessions total, (n)	21.8 ± 13.8	22.1 ± 11.4	21.8 ± 14.3	.61
Duration, (wks)	20.5 ± 11.2	20.9 ± 10.2	20.4 ± 11.4	.73
Therapy end after 13 weeks	60 (30.9)	12 (37.5)	48 (29.4)	.38
Hand splints				.03†
CAM	176 (89.8)	33 (100)	143 (87.7)	
MSS	20 (10.2)	0	20 (12.3)	
PIP extension	81 (42.2)	17 (51.5)	64 (39.3)	.47
Anesthesia				.05†
Plexus	98 (50.3)	19 (57.6)	79 (48.5)	
General	56 (28.7)	14 (42.4)	42 (25.8)	
Local	41 (21.0)	0	41 (25.2)	

∗ Unless specified otherwise.

† Statistically significant difference between zones (*P* ≤ .05). These variables were entered into the regression models.

Recovery of ROM expressed in percentages to the contralateral hand was generally greater when the MCP joint was included in the TAM calculations (Table 3). Range of motion significantly improved over time in both zones 2 and 3 (*P* < .001 to .01) (Table 4). The good-to-excellent TAM scores at week 26, according to the Tang and American Society for Surgery of the Hand classification, were 90% and 95% in zone 3 and 76% and 89% in zone 2 (Table 5).

**Table 3:** Mean Scores of Outcome Measurements

Outcome Measurements	Zone 3			Zone 2		
	Mean ± SD	Mean ± SD	Mean ± SD	Mean ± SD	Mean ± SD	Mean ± SD
	6 Wks	13 Wks	26 Wks	6 Wks	13 Wks	26 Wks
TAM scores (ASSH)∗						
Injured hand	148.0 ± 53.6	213.8 ± 54.9	251.4 ± 41.7	167.1 ± 47.1	207.4 ± 45.4	225.1 ± 40.2
Contralateral	281.5 ± 20.2	281.5 ± 20.2	281.5 ± 20.2	277.9 ± 31.0	277.9 ± 31.0	277.9 ± 31.0
Recovery, (%)‖	52.6	76.0	89.3	60.1	74.5	1.0
TPM scores (ASSH)∗						
Injured hand	235.6 ± 40.9	269.2 ± 35.2	293.8 ± 32.0	237.6 ± 36.5	262.7 ± 36.9	272.5 ± 36.1
TAM scores (Tang)†						
Injured hand	87.3 ± 45.9	129.3 ± 45.2	154.2 ± 32.5	84.8 ± 36.8	109.6 ± 39.9	125.6 ± 37.4
Contralateral	184.1 ± 18.0	184.1 ± 18.0	184.1 ± 18.0	178.7 ± 23.4	178.7 ± 23.4	178.7 ± 23.4
Recovery, (%)‖	47.4	70.3	83.8	47.5	61.3	70.3
TPM scores (Tang)†						
Injured hand	155.4 ± 25.3	174.4 ± 19.4	184.5 ± 20.8	136.6 ± 28.0	155.8 ± 30.6	162.9 ± 33.7
DASH scores‡						
Total	0	18.4 ± 15.1	9.2 ± 9.9	0	17.7 ± 14.4	8.4 ± 11.5
Sport	0	37.9 ± 28.1	14.5 ± 16.0	0	33.3 ± 30.1	11.3 ± 17.7
Work	0	23.9 ± 32.8	9.1 ± 13.4	0	23.6 ± 28.4	9.0 ± 16.4
Satisfaction scores§						
Injured hand	0	6.8 ± 2.1	8.0 ± 1.4	0	6.9 ± 1.9	8.0 ± 1.6
Hand strength (kg)						
Injured hand	0	21.9 ± 12.4	33.0 ± 12.7	0	23.9 ± 10.4	33.3 ± 11.2
Contralateral	0	41.1 ± 13.6	41.1 ± 13.6	0	37.1 ± 11.6	37.1 ± 11.6
Recovery, (%)‖	0	53.7	80.4	0	64.3	89.7

TPM, total passive motion.

∗ Sum of MCP + PIP + DIP joint ROM; norm value = 260°.

† Sum of PIP + DIP joint ROM; norm value = 175°.

‡ DASH score scaled on a 0–100 scale. A higher score indicates greater disability.

§ Satisfaction with the injured hand score scaled on a 0–10 Likert scale. A higher score indicates greater satisfaction.

‖ Presents the ROM and strength of the contralateral hand expressed in percentages to the injured hand.

**Table 4:** Improvement in Outcome Measurements 6, 13, and 26 Weeks After Surgery Within and Between Zones of Injury

Outcome Measurements	Zone 3		Zone 2		Both Zones		
	Within-Group Change¶		Within-Group Change¶		Between-Group Differences#		
	6–13 Wks	13–26 Wks	6–13 Wks	13–26 Wks	6 Wks	13 Wks	26 Wks
TAM scores (ASSH)∗							
Mean (95% CI)	−65.8 (−91.3, −40.3)	−37.6 (−59.5, −15.8)	−39.9 (−49.9, −30.0)	−18.0 (−26.4, −9.7)	19.2 (2.1, 36.3)	−6.7 (−23.1, 9.6)	−26.3 (6.1, −38.2)
*P* value (two-tailed)	<.001∗∗	<.001∗∗	<.001∗∗	<.001∗∗	.03∗∗	.42	<.001∗∗
Effect size, (r) †	−0.84	−0.54	−0.62	−0.39	0.40	−0.14	−0.65
TPM scores (ASSH)∗							
Mean (95% CI)	−33.6 (−46.6, −20.7)	−24.7 (−36.1, −13.2)	−25.2 (−32.7, −17.7)	−9.8 (−16.0, −3.6)	2.0 (−10.1, 14.1)	−6.4 (−18.2, 5.4)	−21.3 (−31.1, −11.5)
*P* value (two-tailed)	<.001∗∗	<.001∗∗	<.001∗∗	.002∗∗	.41	.86	.27
Effect size, (r) †	−0.83	−0.81	−0.55	−0.30	0.05	−0.17	−0.60
TAM scores (Tang)‡							
Mean (95% CI)	−42.1 (−63.3, −20.9)	−24.8 (−43.2, −6.5)	−24.7 (−33.1, −16.4)	−16.0 (−23.7, −8.4)	−2.4 (7.8, −17.8)	−19.8 (7.2, −33.9)	−28.6 (5.7, −39.8)
*P* value (two-tailed)	<.001∗∗	.01∗∗	<.001∗∗	<.001∗∗	.76	.006∗∗	<.001∗∗
Effect size, (r) †	−0.66	−0.38	−0.45	−0.35	−0.06	−0.48	−0.81
TPM scores (Tang)‡							
Mean (95% CI)	−19.0 (−26.3, −11.7)	−10.1 (−16.4, −3.7)	−19.2 (−57.4, 18.9)	−7.1 (−12.9, −1.3)	−18.8 (−59.9, 22.3)	−18.5 (−25.7, −11.4)	−21.5 (−28.8, −14.3)
*P* value (two-tailed)	<.001∗∗	.002∗∗	.27	.02∗∗	.76	.02∗∗	.04∗∗
Effect size, (r) †	−0.78	−0.53	−0.37	−0.26	−0.47	−0.64	−0.71
DASH total scores§							
Mean (95% CI)	0	9.3 (3.0, 15.5)	0	9.3 (5.8, 12.9)	0	−0.7 (−6.8, 5.4)	−0.8 (−6.0, 4.5)
*P* value (two-tailed)	0	<.001∗∗	0	<.001∗∗	0	.82	.78
Effect size, (r) †	0	0.68	0	0.64	0	−0.09	0.05
DASH sport scores§							
Mean (95% CI)	0	23.4 (0.9, 45.9)	0	22.0 (12.9, 31.2)	0	−4.6 (−18.7, 9.5)	−3.2 (−19.5, 13.1)
*P* value (two-tailed)	0	.04∗∗	0	<.001∗∗	0	.52	.67
Effect size, (r) †	0	0.99	0	0.72	0	−0.08	−0.10
DASH work scores§							
Mean (95% CI)	0	14.9 (−8.0, 37.8)	0	14.5 (7.9, 21.2)	0	−0.4 (−18.6, 17.9)	−0.02 (−12.8, 12.8)
*P* value (two-tailed)	0	.20	0	<.001∗∗	0	.97	˃.99
Effect size, (r) †	0	0.64	0	0.63	0	−0.02	0.13
Satisfaction scores‖							
Mean (95% CI)	0	−1.1 (−2.3, 0.01)	0	−1.1 (−1.7, −0.4)	0	0.1 (−0.8, 1.0)	0.02 (−0.9, 1.0)
*P* value (two-tailed)	0	.05∗∗	0	<.001∗∗	0	.84	.97
Effect size, (r) †	0	−0.61	0	−0.44	0	−0.03	−0.12
Hand strength (kg)							
Mean (95% CI)	0	−11.1 (−18.8, −3.5)	0	−9.4 (−12.2, −6.7)	0	2.0 (−2.4, 6.4)	−4.5 (−11.0, 2.1)
*P* value (two-tailed)	0	.01∗∗	0	<.001∗∗	0	.37	.93
Effect size, (r) †	0	−0.99	0	−0.74	0	0.23	−0.38

TPM, total passive motion.

∗ Sum of MCP + PIP + DIP joint ROM; norm value = 260°.

† Cohen’s d, where r = 0.10 is a small, r = 0.30 is a medium, and r = 0.50 is a large clinical effect.

‡ Sum of PIP + DIP joint ROM; norm value = 175°.

§ DASH score scaled on a 0–100 scale. A higher score indicates greater disability. The MID ranges from 10.8 to 18.8 points.

‖ Satisfaction with the injured hand on a 0–10 Likert scale. A higher score indicates greater satisfaction. The MID ranged from 1.2 to 1.8 points.

¶ Paired *t* test.

# Independent-sample *t* test; *P* value ≤ .05 for all outcome measurements.

∗∗ Statistically significant differences (*P* ≤ .05)

**Table 5:** Classification of Flexor Tendon Total Active Motion Recovery

TAM Scores Graded According to Tang (2013)							A
Score: [(PIP flexion + DIP flexion) **–** (PIP extension deficit + DIP extension deficit)] × 100/175°							
Zone				Outcomes of Function in % Return of Motion			
	Time	Total	Excellent	Good	Fair	Poor	Failure
	Wks	Fingers (n)	90%–100%	70%–89%	50%–69%	30%–49%	<30%
Zone 3	6	39	1	7	16	6	9
	13	39	14	10	8	4	3
	26	39	13	22	2	2	0
Zone 2	6	174	5	16	43	79	31
	13	174	18	33	85	22	16
	26	174	15	117	26	13	3

TAM Scores Graded According to the ASSH (2000)						**B**
Score: [(MCP flexion + PIP flexion + DIP flexion) **–** (MCP extension deficit + PIP extension deficit + DIP extension deficit)] × 100/260°						
Zone				Outcomes of function in % return of motion		
	Time	Total	Excellent	Good	Fair	Poor
	Wks	Fingers (n)	100%	˃75%	<75%	<50%
Zone 3	6	39	1	6	20	12
	13	39	11	16	10	2
	26	39	13	24	2	0
Zone 2	6	174	2	45	99	28
	13	174	19	106	38	11
	26	174	17	137	19	1

**Figure 2:**
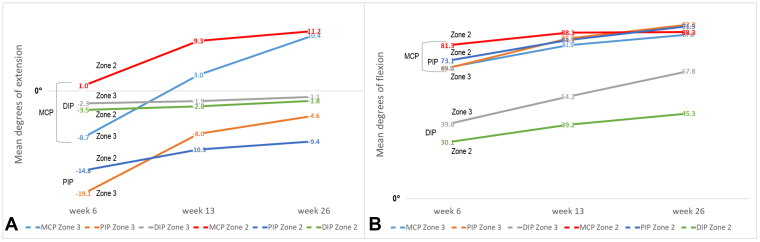
Recovery of finger ROM in degrees in zones 3 and 2 flexor tendon injuries. **A** Means of active finger extension in degrees over time. The negative degrees (<0°) indicate an extension deficit, and the positive degrees (>0°) a hyperextension. **B** Means of active finger flexion in degrees (0°–100°) over time. The MID ranges between 18° to 24° for the MCP, 12° to 15° for the PIP, and 14° to 18° for the DIP joints.

All joints had an active finger extension deficit in week 6, except for the MCP joint in zone 3, and improved over time (Fig. 2A). The DIP and PIP joints did not recover to full extension until week 26 in both zones. MCP flexion in zone 2 was greater at week 6 and smaller after 26 weeks than in zone 3 (Fig. 2B). The PIP joints recovered similarly between zones. The DIP joint always had greater flexion in zone 3 than zone 2. From week 6 to 13, all finger joints made clinically relevant changes, except for the MCP (mean difference 15.2°) and DIP (mean difference 9.8°) joints in zone 2 (Fig. 2). From week 13 to 26, only the DIP joint in zone 3 achieved a MID of 14.4°. All other finger joints continued improving, but they were not clinically relevant.

**Figure 3:**
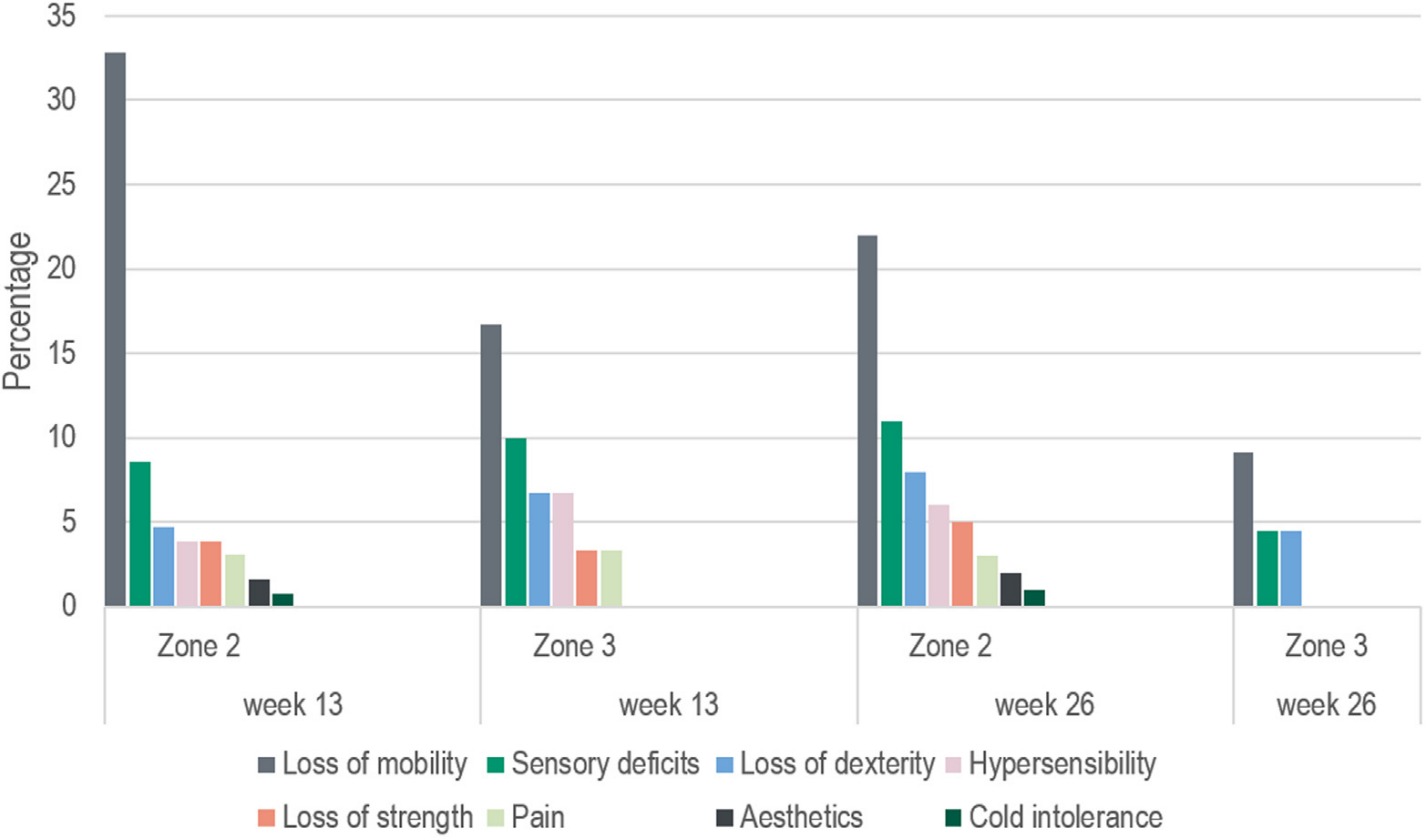
Reasons for dissatisfaction with the hand.

Hand strength recovered to 53.7% and 64.3% of the contralateral hand in zones 3 and 2 at week 13, and continued to improve to 80.4% and 89.7% after 26 weeks, respectively (Table 3). These correspond to statistically significant improvements over time (*P* < .001 to.01) with large effect sizes (r = −0.99 to −0.74) for both zones of injury (Table 4). Between-zone differences were small and statistically nonsignificant (*P* = .37 to .93). Patient satisfaction with their injured hand was generally good to high (Table 3), with no statistically significant differences between the zones of injury (week 13, *P* = .84; week 26, *P* = .97). The most frequent reasons for dissatisfaction were loss of finger mobility in both zones at weeks 13 and 26, followed by sensory deficits and loss of dexterity (Fig. 3). Patient-rated upper-extremity disability and symptoms were generally low as measured with the DASH questionnaire (Table 3). Within-group changes were statistically significant in both zones (*P* < .001) with medium to large effect sizes (zone 3, r = −0.61; zone 2, r = −0.44) (Table 4). There were no relevant between-zone differences in DASH scores at any time point (week 13, 0.1 point; week 26, 0.02 points) (Table 4).

## Discussion

This retrospective cohort study presents the results of 39 primary tendon repairs in zone 3 and compares clinical outcomes with 174 repairs in zone 2. Both patient groups were homogenous in most demographic, injury, therapy, and surgery characteristics, representing, on average, the predestined patient group for flexor tendon injuries that are described in other cohort studies.^4^^,^^10^

Our results showed that zone 3 injuries achieved better results than zone 2 injuries in the primary outcome ROM. Specifically, TAM scores were significantly better when the MCP joint was included at weeks 6 and 26 (American Society for Surgery of the Hand method) and when the MCP joint was excluded at weeks 13 and 26 (Tang method). The differences in Tang scores can be explained by the better active DIP flexion and PIP extension in zone 3 (Fig. 2) and the greater passive Tang scores in zone 3 (mean: 184.5° in zone 3 and 162.9° in zone 2; Table 3). Zone 2 injuries are more prone to flexion contractures in the PIP joint than zone 3 injuries, which have been established as difficult to resolve.^34^ Therefore, tendon-gliding exercises and soft tissue stretching are crucial clinical interventions to address potential adhesions and to reduce potential joint contractures to a minimum. Novel early active motion protocols for zone 2 injuries, such as the Relative Motion Flexion regimen, allowing restricted use of the injured finger from week 1 onward, could further support better ROM in the finger joints.^35^ Although patients after a zone 2 injury had slightly better recovery of hand strength over time than zone 3 patients, differences were statistically nonsignificant between zones of injury. The same trend was observed for patient satisfaction and the DASH scores, with significant changes over time in both zones but not between zones of injury (Table 4). Patient satisfaction with their injured hand was generally good to high (Table 3), leaving little room for clinically important changes over time (−1.1 points for both zones of injury) (Table 4). In our study, the DASH scores were already low at week 13 (Table 3) compared with the normative DASH values for blue (mean 15.6 scores) and white-collar workers (mean 9.7 scores).^36^ This raises the question of whether DASH is the right outcome measurement for this patient population.^37^^,^^38^ For example, the Michigan Hand Outcomes Questionnaire, being slightly more sensitive to functional changes in patients with hand injuries,^39^ might be more suitable for flexor tendon injuries. Therefore, we also included the Michigan Hand Outcomes Questionnaire in our flexor tendon registry, although currently with too little data collected to be analyzed.

This study had some limitations. Missing data was an issue in this retrospective cohort study, with approximately one-third of the clinical data missing in both zones at week 26. We corrected for missing data using multiple imputations.^30^ The imbalance in sample size between zones of injury was a challenge. We could not match groups, eg, by propensity score matching, because our patients all had the same interventions and measurements.^40^ We addressed this limitation by conducting univariate analysis for each demographic, injury, surgery, and therapy characteristic. The inclusion of multiple finger injuries and two early active rehabilitation protocols may have influenced clinical outcomes. Future studies should further examine the choice of rehabilitation protocol and the effect of multiple finger injuries on clinical outcomes after flexor tendon repairs.

## Supplementary Material

Supplementary Tables 1 and 2 Non-responder analysis.
