# Supplementary material for: Outcomes of Primary Flexor Tendon Repairs in Zones 2 and 3: A Retrospective Cohort Study
Source: J Hand Surg Glob Online. 2023 May 18;5(4):445–53. doi: 10.1016/j.jhsg.2023.03.016 (PMC10382891; doi:10.1016/j.jhsg.2023.03.016)
Supplement: Supplementary Tables 1 and 2 — Non-responder analysis. [file mmc1.pdf]

Supplementary Table 1: Non-responder analysis.

	Non-responder analysis					
	Zone 3			Zone 2		
Demographics	Primary outcome at 26 weeks present	Primary outcome at 26 weeks absent	p-value (exact 2-tailed)	Primary outcome at 26 weeks present	Primary outcome at 26 weeks absent	p-value (exact 2-tailed)
Patients, n (%)	21 (63.6)	12 (36.4)		93 (57.1)	70 (42.9)	
Mean age ± SD (years)	35.3 ± 13.3	35.5 ± 11.8	.97	36.8 ± 13.8	35.9 ± 15.7	.68
Gender, n (%)			.43			.87
Men	14 (66.7)	10 (83.3)		62 (66.7)	48 (68.6)	
Women	7 (33.3)	2 (16.7)		31 (33.3)	22 (31.4)	
Work status, n (%)			.07			.62
Blue collar worker	10 (47.6)	10 (83.3)		57 (61.3)	46 (65.7)	
White collar worker	11 (52.4)	2 (16.7)		36 (38.7)	24 (34.3)	
Type of injury, n (%)			.76			.18
Clean cut	18 (85.7)	10 (83.3)		81 (87.1)	61 (87.1)	
Mild crush	2 (9.5)	2 (16.7)		6 (6.5)	8 (11.4)	
Moderate crush	1 (4.8)	0		6 (6.5)	1 (1.4)	
Injured hand, n (%)			.72			
Dominant	10 (47.6)	7 (58.3)		43 (46.2)	40 (57.1)	.21
Non-dominant	11 (52.4)	5 (41.7)		50 (53.8)	30 (42.9)	
Injured finger, n (%)	25 (64.1)	14 (35.9)	.67	99 (55.6)	75 (68.0)	.70
Dig 2	5	4		36	22	
Dig 3	4	2		15	12	
Dig 4	10	3		16	11	
Dig 5	6	5		32	30	
Multiple fingers, n	4	2	.63	5	4	.93
Mean time injury to surgery ± SD (days)	1.4 ± 1.5	1.1 ± 2.1	.64	2.6 ± 4.4	3.5 ± 5.3	.23
Pulley venting, n (%)	13 (52.0)	4 (28.6)	.19	55	51	.07
Mean TAM ± SD, week 6 [a)]	83.1 ± 38.9	93.9 ± 50.0	.46	82.9 ± 33.8	86.7 ± 34.1	.46
Mean TAM ± SD, week 6 [b)]	141.2±46.7	158.9±55.3	.30	162.9±42.4	174.7±44.0	.08

N: number; SD: standard deviation; dig: digit; TAM: total active motion score in degrees ([a)] Tang method; [b)] ASSH method)
Participants with a primary outcome at 26 weeks were compared to those without a ROM measurement at 26 weeks using Fisher exact tests, Chi-square tests and independent sample t-tests.

Supplementary Table 2. Overview of original and pooled outcome measurements data.

	Zone 3						Zone 2					
	**Original data**			**Pooled data**			**Original data**			**Pooled data**		
	Mean ± SD			Mean ± SD			Mean ± SD			Mean ± SD		
Outcome measurements	6 weeks	13 weeks	26 weeks	6 weeks	13 weeks	26 weeks	6 weeks	13 weeks	26 weeks	6 weeks	13 weeks	26 weeks
**TAM scores (ASSH)** [a]												
Injured hand	147.9±53.6	213.8±54.9	251.2±41.7	148.0±53.6	213.8±54.9	251.4±41.7	167.1±47.1	207.0±45.4	225.0±40.2	167.1±47.1	207.4±45.4	225.1±40.2
Contralateral	281.5±20.2	281.5±20.2	281.5±20.2	281.5±20.2	281.5±20.2	281.5±20.2	277.3±31.0	277.3±31.0	277.3±31.0	277.9±31.0	277.9±31.0	277.9±31.0
Recovery,% [e]	52.6	76.0	89.2	52.6	76.0	89.3	60.3	74.8	81.1	60.1	74.5	81.0
**TPM scores (ASSH)** [a]												
Injured hand	235.8±40.9	269.1±35.2	293.5±32.0	235.6±40.9	269.2±35.2	293.8±32.0	237.6±36.5	262.8±36.9	272.5±36.1	237.6±36.5	262.7±36.9	272.5±36.1
**TAM scores (Tang)** [b]												
Injured hand	87.2±45.9	129.3±45.2	154.0±32.5	87.3±45.9	129.3±45.2	154.2±32.5	84.8±36.8	109.6±39.9	124.3±37.4	84.8±36.8	109.6±39.9	125.6±37.4
Contralateral	184.0±18.0	184.0±18.0	184.0±18.0	184.1±18.0	184.1±18.0	184.1±18.0	178.3±23.4	178.3±23.4	178.3±23.4	178.7±23.4	178.7±23.4	178.7±23.4
Recovery,% [e]	47.4	70.3	83.7	47.4	70.3	83.8	47.6	61.5	69.7	47.5	61.3	70.3
**TPM scores (Tang)** [b]												
Injured hand	155.4±25.3	174.3±19.4	184.4±20.8	155.4±25.3	174.4±19.4	184.5±20.8	142.5±28.0	155.9±30.6	162.2±33.7	136.6±28.0	155.8±30.6	162.9±33.7
**DASH scores** [c]												
Total		18.4 ± 15.1	8.4 ± 9.9		18.4 ± 15.1	9.2 ± 9.9		17.2 ± 14.4	9.0 ± 11.5		17.7 ± 14.4	8.4 ± 11.5
Sport		33.0 ± 28.1	12.0 ± 16.0		37.9 ± 28.1	14.5 ± 16.0		30.7 ± 30.1	10.2 ± 17.7		33.3 ± 30.1	11.3 ± 17.7
Work		23.1 ± 32.8	7.1 ± 13.5		23.9 ± 32.8	9.1 ± 13.5		22.6 ± 28.4	9.2 ± 16.4		23.6 ± 28.4	9.0 ± 16.4
**Satisfaction scores** [d]												
Injured hand		7.0 ± 2.1	8.1 ± 1.5		6.8 ± 2.1	8.0 ± 1.4		6.9 ± 1.9	7.9 ± 1.6		6.9 ± 1.9	8.0 ± 1.6
**Hand strength, kg**												
Injured hand		21.3 ± 12.4	35.8 ± 12.7		21.9 ± 12.4	33.0 ± 12.7		23.8 ± 10.4	33.3 ± 11.2		23.9 ± 10.4	33.3 ± 11.2
Contralateral		40.9 ± 13.6	40.9 ± 13.6		41.1 ± 13.6	41.1 ± 13.6		37.1 ± 11.6	37.1 ± 11.6		37.1 ± 11.6	37.1 ± 11.6
Recovery,% [e]		52.1	87.5		53.7	80.4		64.2	89.9		64.3	89.7

TAM: total active motion / TPM: total passive motion / DASH: Disability of Arm Shoulder Hand questionnaire / SD: Standard deviation / %: percentage / kg: kilogram

[a] Sum of MCP + PIP + DIP joint range of motion; norm value = 260°

[b] Sum of PIP + DIP joint range of motion; norm value = 175°

[c] DASH score scaled on a 0-100 scale. A higher score indicates greater disability.

[d] Satisfaction with injured hand score scaled on a 0-10 Likert scale. A higher score indicates greater satisfaction.

[e] Presents the range of motion and strength of the injured hand expressed in percentages to the contralateral hand
